# Stay-Green QTLs Response in Adaptation to Post-Flowering Drought Depends on the Drought Severity

**DOI:** 10.1155/2018/7082095

**Published:** 2018-11-18

**Authors:** Nasrein Mohamed Kamal, Yasir Serag Alnor Gorafi, Hisashi Tsujimoto, Abdelbagi Mukhtar Ali Ghanim

**Affiliations:** ^1^Biotechnology and Biosafety Research Center, Agricultural Research Corporation, P.O. Box 30, Shambat, Khartoum North, Sudan; ^2^Arid Land Research Center, Tottori University, 1390 Hamasaka, Tottori 680-0001, Japan; ^3^Agricultural Research Corporation, P.O. Box 126, Wad Medani, Sudan; ^4^Plant Breeding and Genetics Laboratory, FAO/IAEA Joint Division of Nuclear Techniques in Food and Agriculture, International Atomic Energy Agency (IAEA), Seibersdorf, Austria

## Abstract

Stay-green trait enhances sorghum adaptation to post-flowering drought. Six stay-green backcross introgression lines (BILs) carrying one or more stay-green QTLs (Stg1-4) and their parents were characterized under non-stress (W_100_: 100% of soil field capacity (FC)) and two levels of post-flowering drought (W_75_: 75% FC; W_50_: 50% FC) in a controlled condition. We aimed to study the response and identify the drought threshold of these QTLs under different levels of post-flowering drought and find traits closely contributing to grain yield (GY) under different drought severity. W_50_ caused the highest reduction in BILs performance. From W_100_ to W_50_, the GY of the recurrent parent reduced by 70%, whereas that of the BILs reduced by only 36%. W_75_ and W_50_ induce different behavior/response compared to W_100_. Harvest index contributed to the GY under the three water regimes. For high GY under drought transpiration rate at the beginning of drought and mid-grain filling was important at W_75_, whereas it was important at mid-grain filling and late-grain filling at W_50_. Stay-green trait can be scored simply with the relative number of green leaves/plants under both irrigated and stress environments. QTL pyramiding might not always be necessary to stabilize or increase the GY under post-flowering drought. The stay-green QTLs increase GY under drought by manipulating water utilization depending on drought severity.

## 1. Introduction

The global population will increase to 9 billion by 2050 and most of the increase will occur in sub-Saharan Africa [1], increasing the risk of food insecurity in this region [2]. Therefore, making plants resilient to the challenges of a water-scarce planet where climate change and global warming threaten food supplies is the major challenge facing the humanity [3]. Drought is perhaps the most important abiotic stress limiting crop productivity in the rain-fed agriculture around the world [4]. Sorghum (*Sorghum bicolor *(L.) Moench) is a grain crop that is well adapted to hot and dry climates. The productivity of sorghum, the major cereal crop grown for food, feed, and fuel, is usually under threat of terminal drought, which is likely to occur in rain-fed environments during grain filling [5]. It is reported that stay-green genotypes that exhibited the ability to retain green leaf area during grain filling under terminal drought produce higher grain yield than the non-stay-green genotypes [6–10]. Over the last 30 years, the stay-green trait has been used in breeding programs for improvement of sorghum terminal drought tolerance [11, 12]. Several sources for stay-green have been identified, including B35, SC56, and E-36 [13–15]. The quantitative trait loci (QTLs) that contribute to the stay-green trait have been mapped in a range of populations, mostly derived from crosses with B35, a derivative of an Ethiopian dura landrace [16–23]. Xu and Sanchez [23, 24] identified four major QTLs in B35, including Stg1 (Stay-green QTL 1) and Stg2 both located on the linkage group SBI-03, Stg3 on SBI-02 and Stg4 on SBI-05. These QTLs explain 20, 30, 16, and 10%, respectively, of the phenotypic variation of the stay-green under post-flowering drought stress. Reddy [25] validated those QTLs reported in earlier studies and indicated that Stg2 and Stg3 were prominent in their expression.

The exact physiological mechanism of stay-green and the role of each individual QTL on the final phenotype of the stay-green genotypes under post-flowering drought stress are still unclear. However, a number of reports increased our understanding of this complicated drought adaptation mechanism. Borrell and Hammer [26, 27] explained the delayed onset and reduced rate of leaf senescence in stay-green genotypes by the high specific leaf nitrogen and nitrogen uptake during grain filling. Harris [28] reported that, under post-flowering water deficit, Stg2, Stg3, and Stg4 near isogenic lines (NILs) exhibited delayed onset of leaf senescence compared with the non-stay-green genotype, RTx7000, while significantly lower rates of leaf senescence in relation to RTx7000 were displayed by all of the stay-green NILs to varying degrees, but particularly by the Stg2 NIL. The Stg1 and Stg4 NILs exhibited greener leaves at flowering relative to RTx7000, indicated by higher SPAD values. Borrell [29] reported that hybrids containing B35, the source of stay-green, have higher transpiration efficiency (TE) than other eight hybrids examined. They suggested that the higher TE was due to increased photosynthetic capacity associated with higher specific leaf nitrogen, rather than reduced stomatal conductance. Vadez [9] studied the effect of different stay-green QTLs on modification of tillering and leaf area at flowering, transpiration efficiency, water extraction, harvest index, and grain yield under both terminal drought and fully irrigated conditions in 29 introgression lines with different stay-green QTLs in two backgrounds. They concluded that StgB and Stg1 modify the TE and water extraction depending on the background. Stay-green QTLs decrease the canopy size before flowering to conserve soil water for use during grain filling; the increased water uptake during grain filling in stay-green NILs relative to the non-stay-green parent RTx7000 resulted in higher biomass production, grain number, and yield [5, 30]. Moreover, Jaegglia [31] explained that tiller leaf area rather than transpiration efficiency, or transpiration per leaf area, was the main driver of weekly transpiration and the reduced pre-flowering water use in stay-green lines. According to Vadez and Borrell [5, 9, 30], the differences in TE are still unexplained and work is ongoing to investigate traits that might be related to leaf conductance aspects. In soils with good water-holding capacity, any water savings during the pre-flowering period increases water availability during the post-flowering period, therefore allowing plants to retain the photosynthetic capacity for longer by ‘staying green' during grain filling [31].

The effect of the stay-green QTLs on modification of plant parameters under different levels of post-flowering drought is not extensively studied. Effect comparison between single QTL and QTL pyramiding under a specific level of drought severity has not been investigated adequately. Previously, we introgressed the stay-green trait into the Sudanese sorghum cultivar ‘Tabat' [32, 33] and evaluated the response of the BC_2_F_4_ stay-green lines under irrigated and drought conditions; we concluded that identification of the drought threshold is needed for better understanding the physiological reactions under specific drought severity. In this study, we analyzed the effect of single or more stay-green QTLs on modification of plant performance under controlled, non-stress condition and two levels of post-flowering drought stress by examining the plant physiological status through direct measurement of the leaves activity under drought. Also, we aimed to identify the level of drought to which those stay-green QTLs can confer drought tolerance. Moreover, we studied traits that mostly contribute to grain yield under different drought severity.

## 2. Materials and Methods

### 2.1. Plant Materials

Six BC_3_F_3_ stay-green backcross introgression lines (BILs) derived from a cross of drought sensitive cultivar ‘Tabat' (abbrev. TAB, the recurrent parent) and stay-green donor B35 were used in this study. The backcross lines were produced at Agricultural Research Corporation, Sudan [32, 33]. Out of the six BILs, four carry a single stay-green QTL (Stg1, Stg2, Stg3, and Stg4, respectively), one BIL carries two QTLs (Stg1+4), and the other BIL carries three QTLs (Stg1+2+4). TAB is an improved Sudanese variety released for irrigated areas [34, 35] and B35 is a partially converted selection of the durra sorghum IS12555 from Ethiopia [13]. There was no big variation in the flowering of the genotypes and all flowered in the range from 70 to 76 days after sowing.

### 2.2. Pot Experiment

A pot experiment was conducted in a glasshouse in the Arid Land Research Center (ALRC), Tottori University (Tottori, Japan; 35°32′N, 134°13′E) from June to November. The pots were filled with 15 kg of Tottori sandy soil. The chemical properties were reported by Fujiyama and Nagai [36]. As reported by Sohail et al. [37], we used three inorganic fertilizers: compound macronutrients N : P : K (16 : 16 : 16) in a rate of 0.4g/Kg (Central Glass Co., Ltd, Tokyo, Japan) and Ca : Mg (21 : 0.6) (Hitachi Chemical Co., Ltd, Tokyo, Japan) at a rate of 0.7g/Kg and micronutrients Mg : Mn : B (8.4 : 0.3 : 0.3) (MC Ferticom Co., Ltd, Tokyo, Japan) at a rate of 0.3g/Kg.

Five seeds were sown per pot, and two weeks after sowing the seedlings were thinned and only one plant was allowed to grow beyond until maturity stage. After flowering, we applied three different drought levels (soil water regimes): W_100_ (100% of soil field capacity (FC); water content was 120 ml/kg soil); W_75_ (75% of soil FC; water content was 90 ml/kg soil) representing moderate drought; and W_50_ (50% of soil FC representing severe drought; water content was 60 ml/kg soil). The experiment was arranged in a completely randomized design with three replications. The position of each pot was randomized and changed weekly in the glasshouse to ensure uniform environmental conditions. Usually, the pots were weighted every day and irrigated with tap water to keep the specific FC; usually, pots in W_100_ were irrigated before reaching the FC of the pots in W_75_, and pots in W_75_ were irrigated before reaching the FC of the pots in W_50_, whereas pots in W_50_ were irrigated before reaching FC of 35%. During the experiment, the average maximum temperature in the glasshouse ranged from 34 to 23°C and the minimum ranged from 19 to 10°C.

### 2.3. Morphological Traits

Days to heading (DTH) were calculated as the number of days between the sowing date and the date when 50% of all the shoots in a pot had fully emerged spikes. At physiological maturity, plant height (PH) was measured in centimeters (cm) from the ground to the tip of the spike in each pot before harvesting. Days to maturity (DTM) were calculated from sowing date to 50% senescence of the spikes. Finally, grain yield per pot (GY) was determined as the weight (grams) of the grain from each pot; Biomass (BM) was determined as the weight (grams) of the aboveground fresh biomass, and harvest index (HI) was calculated using the formula

(1) $$\begin{array}{c} HI=\frac{Grain\;\;yield}{above\;\;ground\;\;biomass}*100. \end{array}$$

Yield susceptibility index (YSI) was calculated according to Fischer and Maurer [37]:

(2) $$\begin{array}{c} YSI=\frac{\left(1–Y/Yp\right)}{D} \end{array}$$

where* Y* is the GY of the genotype at drought,* Yp* is the mean GY of the genotypes at control, and* D *(stress intensity) = 1 –* X*/*Xp*, where* X* is the mean* Y* of all genotypes and* Xp* is the mean* Yp* of all genotypes. Genotypes were classified as highly tolerant (YSI ≤ 0.50), moderately tolerant (0.50 < YSI ≤ 1.00), or sensitive (YSI > 1.00) to drought [38, 39].

### 2.4. Leaf Measurement for Chlorophyll Content and Relative Number of Green Leaves/Plants

Detailed leaf observations were made on three replicates in each treatment. Fully expanded and senesced leaf number was recorded as described by Hammer [40] at one week before drought (WD), mid-grain filling (GF), and maturity (M).

A leaf was considered fully expanded when its ligule became visible above the enclosing sheath of the previous leaf. A leaf was considered senesced when more than 50% of its area turned yellow. Relative number of green leaves/plants (GN) was calculated as

(3) $$\begin{array}{c} \frac{\text{a number of green leaves per plant}}{\text{total number of leaves per plant}}*100. \end{array}$$

Leaf area of each individual fully expanded leaf was estimated nondestructively from the product of its length, greatest width, and a shape factor of 0.57, which was established by regressing the product of width and length of a leaf against its actual leaf area measured destructively at the end of the experiment. These estimates of individual leaf sizes, combined with observations of fully expanded and senesced leaves, allowed the estimation of green leaf area [41].

For the chlorophyll content (SPAD) data was represented as relative chlorophyll content (RCC) to ease the explanation and understanding of the degradation of leaf chlorophyll (senescence). Chlorophyll content was measured by the chlorophyll meter SPAD-502 (Konica Minolta). The arbitrary SPAD values can be translated into the actual value of total chlorophyll/unit area (mg cm^−2^) using the equation: Chlorophyll content= SPAD values x 0.003 — 0.048, as described by Xu [23].

### 2.5. Leaf Gas Exchange Measurement

The fully expanded second leaf from the apex position and flag leaf were used for measurements. The photosynthesis rate (PR) and transpiration rate (TR) were measured using LI-6400 portable photosynthesis system (LI-COR Bioscience, Lincoln, NE, USA), at three growth stages: WD, GF, and late-grain filling (LGF) during sunny days. During measurement the chamber temperature was 25°C, the reference CO_2_ concentration was 400 *μ*mol mol^−1^, the relative humidity was approximately 25%, and the irradiance was 1200 *μ*mol m^−2^ s^−1^.

### 2.6. Statistical Analysis

Two-way ANOVA was performed to assess the effect of genotype (G), drought treatment (W), and genotype-by-drought treatment (G×W) interaction for the different traits measured using GenStat version 17. The ANOVA was followed by Fisher's protected least significant difference (PLSD) test at* P* < 0.05. PCA analysis was performed using STAR software (STAR, version 1.4., International Rice Research Institute, Los Baños, Philippines; http://bbi.irri.org/products). Simple linear regression was performed using a linear regression model.

## 3. Results

### 3.1. The Drought Threshold of the Stay-Green BILs

The drought treatment effect was significant for all traits studied under W_100_, W_75_, and W_50_ (*P*<0.0001). In all traits except BM, PH, PR, and TR at WD, W_100_ had the highest mean values compared to W_75_ and W_50_ with the latter being the lowest (Tables 1 and 2). In the case of BM, W_100_ and W_75_ did not differ significantly, whereas, in the case of PH, W_75_ and W_50_ did not differ significantly. These findings indicated clearly that W_50_ as drought treatment was more severe than W_75_. From W_100_ to W_50_, the GY of the recurrent parent reduced by 70% whereas that of the BILs reduced by only 36%. The reduction from W_100_ to W_50_ did not exceed 50% in all traits except GLA and PR at LGF. Nevertheless, the BILs showed clear stay-green expression under W_75_ (moderate drought) (Tables 1 and 2).

### 3.2. The Effect of the Introgressed QTLs under No Drought at W_100_

**Figure 1:**
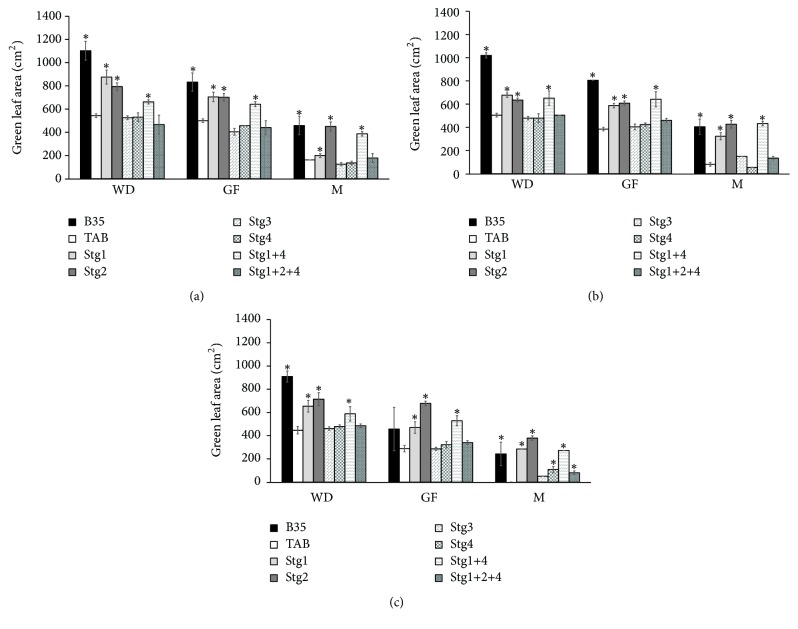
Green leaf area (GLA) at one week before drought (WD), mid-grain filling (GF), and maturity (M) under control W_100_ (a), 75% field capacity W_75_ (b), and 50% filed capacity W_50_ (c) of the six stay-green sorghum introgression lines evaluated with their parents under W_100_, W_75_, and W_50_ of soil field capacity. Asterisks indicate significant difference from Tabat (TAB) (*P* < 0.05, Fisher's PLSD test).

**Figure 2:**
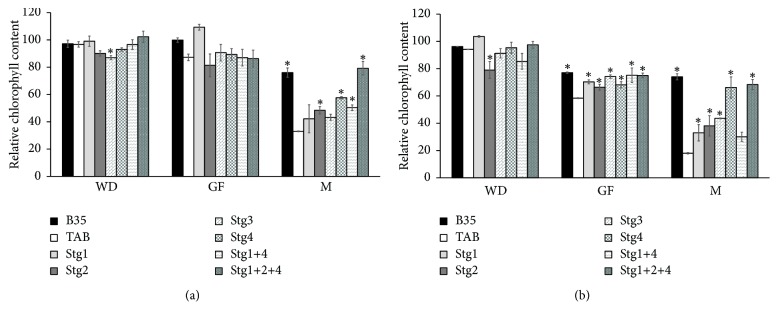
Relative chlorophyll content (RCC) at one week before drought (WD), mid-grain filling (GF), and maturity (M) under control W_100_ (a), 75% field capacity W_75_ (b), and 50% filed capacity W_50_ (b) of the six stay-green sorghum introgression lines evaluated with their parents under W_100_, W_75_, and W_50_ of soil field capacity. Asterisks indicate significant difference from Tabat (TAB) (*P* < 0.05, Fisher's PLSD test).

**Figure 3:**
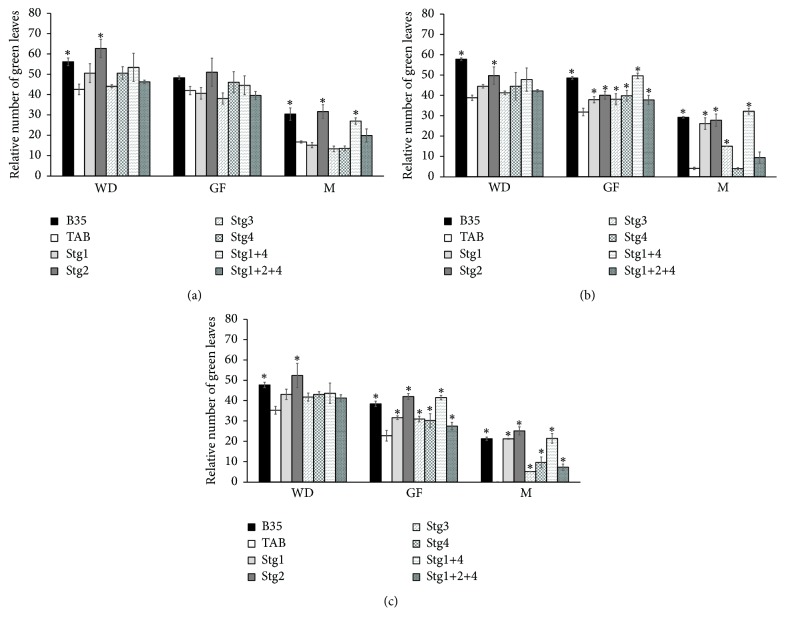
Relative number of green leaves/plants (GN) at one week before drought (WD), mid-grain filling (GF), and maturity (M) under control W_100_ (a), 75% field capacity W_75_ (b), and 50% filed capacity W_50_ (c) of the six stay-green sorghum introgression lines evaluated with their parents under W_100_, W_75_, and W_50_ of soil field capacity. Asterisks indicate significant difference from Tabat (TAB) (*P* < 0.05, Fisher's PLSD test).

**Figure 4:**
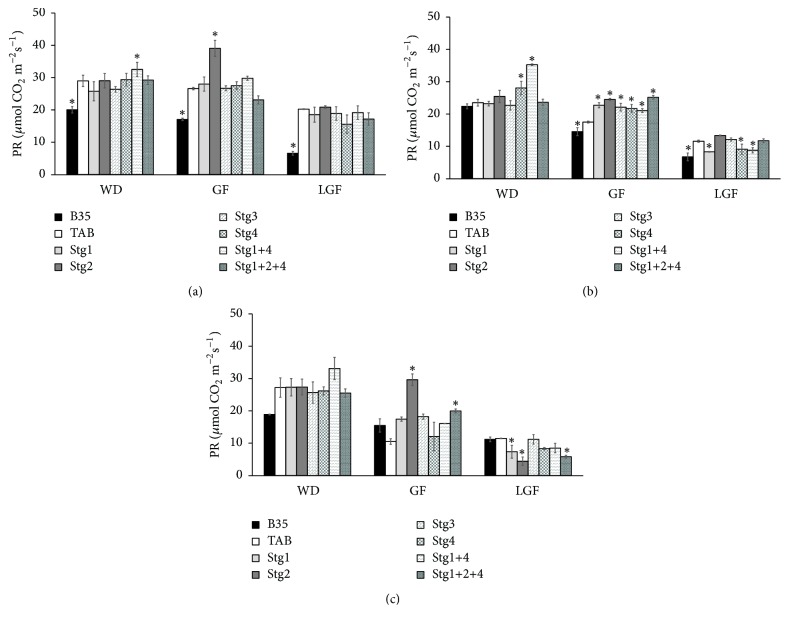
Photosynthesis rate (PR) at one week before drought (WD), mid-grain filling (GF), and late-grain filling (LGF) under control W_100_ (a), 75% field capacity W_75_ (b), and 50% filed capacity W_50_ (c) of the six stay-green sorghum introgression lines evaluated with their parents under W_100_, W_75_, and W_50_ of soil field capacity. Asterisks indicate significant difference from Tabat (TAB) (*P* < 0.05, Fisher's PLSD test).

**Figure 5:**
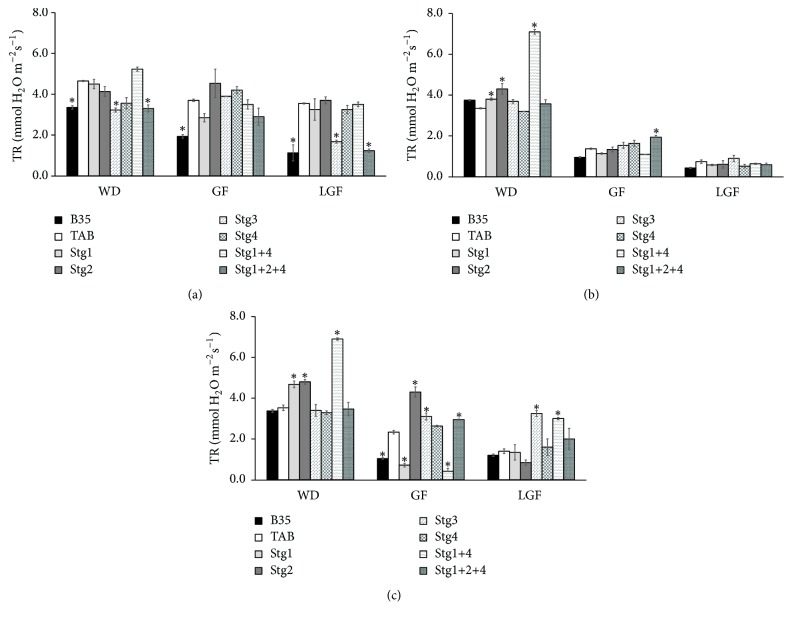
Transpiration rate (TR) at one week before drought (WD), mid-grain filling (GF), and late-grain filling (LGF) under control W_100_ (a), 75% field capacity W_75_ (b), and 50% filed capacity W_50_ (c) of the six stay-green sorghum introgression lines evaluated with their parents under W_100_, W_75_, and W_50_ of soil field capacity. Asterisks indicate significant difference from Tabat (TAB) (*P* < 0.05, Fisher's PLSD test).

Generally, all the BILs with the different QTLs were comparable to or even better than TAB in their performance at W_100_ (Table 1 and Figures 1–5). However, BIL Stg1+4 had lower GY and HI, and BIL Stg3 had lower BM than TAB (Table 1). BILs Stg1, Stg2, and Stg1+4 had higher GLA than TAB at all stages (Figure 1(a)). BILs Stg3 and Stg1+2+4 had lower TR than TAB at WD and LGF, whereas at GF all BILs were comparable to TAB, but BIL Stg2 showed a substantial increase in TR (Figure 5(a)).

### 3.3. The Effect of the Introgressed QTLs under Stress Conditions at W_75_ and W_50_

Overall, the performance of the stay-green BILs was better than that of the recurrent parent TAB under both W_75_ and W_50_ (Table 1, Figures 1–5).

All the BILs had higher GY than TAB under both W_75_ and W_50_ except BILs Stg2 and Stg4 (Table 1). BIL Stg2 had lower GY than TAB under both W_75_ and W_50_, whereas BIL Stg4 had comparable GY to that of TAB under W_75_. TAB and BIL Stg2 showed the highest reduction in GY from W_100_ to W_75_ and W_50_, whereas B35 and Stg3 showed the lowest reduction. These lines showed rather substantially higher GY under W_75_ than that under W_100_. This may explain the ability of the stay-green genotypes to increase translocation efficiency under a specific level of drought.

The other Stg lines were intermediate between their parents in their reduction (Table 1). The stay-green QTLs improved the GY of TAB under drought by different magnitudes, the GY of TAB improved by 25.1% with Stg1+4 to 54.6% with Stg3 under W_75_, and by 87.5% with Stg1 to 186.1% with Stg3 under W_50_ (Table 1).

To show the degree of drought tolerance conferred by each of the stay-green QTLs, we calculated the YSI at both W_75_ and W_50_. TAB showed 1.3 and 1.6 YSI at W_75_ and W_50_, respectively, and BIL Stg2 showed 2.9 and 2.3 at W_75_ and W_50_. These were classified as sensitive (YSI > 1.00). BIL Stg3 showed -0.36 and -0.08, BIL Stg1+4 showed 0.55 and 0.56, and BIL Stg1+2+4 showed 0.22 and 0.50, under W_75_ and W_50_, respectively. These were classified as highly tolerant (YSI ≤ 0.50). BIL Stg4 showed 0.73 and 0.67 and was regarded as moderately tolerant (0.50 < YSI ≤ 1.00). BIL Stg1 was tolerant at W_75_ (0.49) but moderately tolerant at W_50_ (0.80).

In BM, BILs Stg1, Stg2, Stg4, and Stg1+4 had higher BM than TAB under both W_75_ and W_50_. BIL Stg3 which had the highest GY under W_50_ had lower BM than TAB and was the least in the ranking of the genotypes at W_50_ (Table 1). Except for BIL Stg2, all of the BILs and their parents showed slight or no reduction in BM from W_100_ to W_75_ and W_50_. BIL Stg2 did not exhibit any reduction and had the highest BM at both W_75_ and W_50_ and was the top in the ranking of the genotypes (Table 1).

In HI, BILs Stg3 and Stg1+2+4 had higher HI than TAB under both W_75_ and W_50_, whereas BILs Stg2 and Stg1+4 had lower HI than TAB under both treatments (Table 1). Among the BILs, Stg3 was the highest with no reduction from W_100_ to W_75_ and W_50_, whereas Stg2 was the lowest with the highest reduction from W_100_ to W_75_ and W_50_ (Table 1).

In PH, BILs Stg3 and Stg1+2+4 had higher PH than TAB at both W_75_ and W_50_ (Table 1). At W_75_, also Stg4 and Stg1+4 had higher PH than TAB. The highest reduction in PH was exhibited by Stg1, whereas Stg1+2+4 did not exhibit any reduction compared to TAB. Stg2 and Stg3 showed a slight reduction (Table 1).

In GLA, under both W_75_ and W_50_ at all stages, Stg1, Stg2, and Stg1+4 had higher GLA than TAB (Figures 1(b) and 1(c)). B35 did not show any reduction, whereas the reduction in TAB, Stg1 and Stg2, was higher than that in the other lines. From W_100_ to W_50_ the reduction in TAB and B35 was higher than that in the other lines. All the lines showed a reduction of GLA from WD to GF with different magnitudes (Figure 1(b)). Line Stg1+4 showed the lowest reduction from WD to GF. From GF to M the reduction was higher than that from WD to GF (Figures 1(b) and 1(c)).

Generally, RCC of TAB reduced with the progress of the drought and was low at W_50_, whereas BILs showed improved RCC than TAB (Figure 2). At WD, all the BILs were comparable to TAB, except BIL Stg3 under W_75_ and BIL Stg2 under W_50_ (Figures 2(a) and 2(b)). At GF, all the BILs were comparable to TAB under W_75_, whereas they had higher RCC than TAB under W_50_ (Figures 2(a) and 2(b)). At M, under W_75_ all BILs except Stg1 and Stg3 had higher RCC than TAB, whereas under W_50_, except Stg1+4, all the BILs had higher RCC than TAB. B35 had the highest RCC and maintained more than 75% of its RCC from WD to M at both W_75_ and W_50_, whereas TAB maintained only 20% of its RCC and showed the highest rate of reduction (Figure 2). On the other hand, BILs showed different reduction magnitudes and maintained higher RCC than TAB under the W_50_ and W_75_ treatments, which indicate their ability to retain more chlorophyll content than TAB.

In GN, all the BILs performed better than TAB (Figure 3). At WD, only BIL Stg2 was higher than TAB under both W_75_ and W_50_, whereas at GF and M, all the BILs were higher than TAB, except Stg4 and Stg1+2+4 at M under W_75_ (Figures 3(b) and 3(c)). Similar to the RCC, all BILs showed less reduction rates compared to TAB, and they were able to partially maintain their GN at M under W_50_ when TAB was completely dry (Figure 3(c)).

In PR, all the BILs and their parent PR decreased with the progress of the drought from W_75_ to W_50_ and from WD to GF and LGF (Figure 4). At WD, the BILs showed comparable PR to that of TAB under both W_75_ and W_50_, except that Stg4 and Stg1+4 under W_75_ had higher PR than TAB (Figure 4(b)). At GF, under W_75_, all the BILs had higher PR than TAB, whereas under W_50_, Stg2 and Stg1+2+4 showed higher PR than TAB (Figures 4(b) and 4(c)). At LGF, some BILs had comparable PR to that of TAB and others showed less PR than that of TAB. At GF the lowest reduction from W_100_ to W_75_ and from W_100_ to W_50_ was observed in line Stg1+2+4 indicating that QTL pyramiding is essential to maintain stable photosynthesis under drought conditions (Figure 4).

As in the PR, the TR decreased with the progress of the drought from W_75_ to W_50_ and from WD to GF and LGF (Figure 5), but interestingly BILs Stg3 and Stg1+2+4 showed the ability to maintain stable TR with the progress of the drought. However, their TR was lower than TAB under W_100_ without drought (Figure 5). At WD, BILs Stg1, Stg2, and Stg1+4 showed higher TR than TAB under both W_75_ and W_50_. At GF, BIL Stg1+2+4 showed higher TR than TAB under both W_75_ and W_50_, and BILs Stg2 and Stg3 were higher than TAB under W_50_. At LGF, under W_75_ Stg3 had substantially higher TR than TAB, whereas, under W_50_, Stg3 and Stg1+4 had higher TR than TAB (Figure 5).

### 3.4. Principal Component Analysis

**Figure 6:**
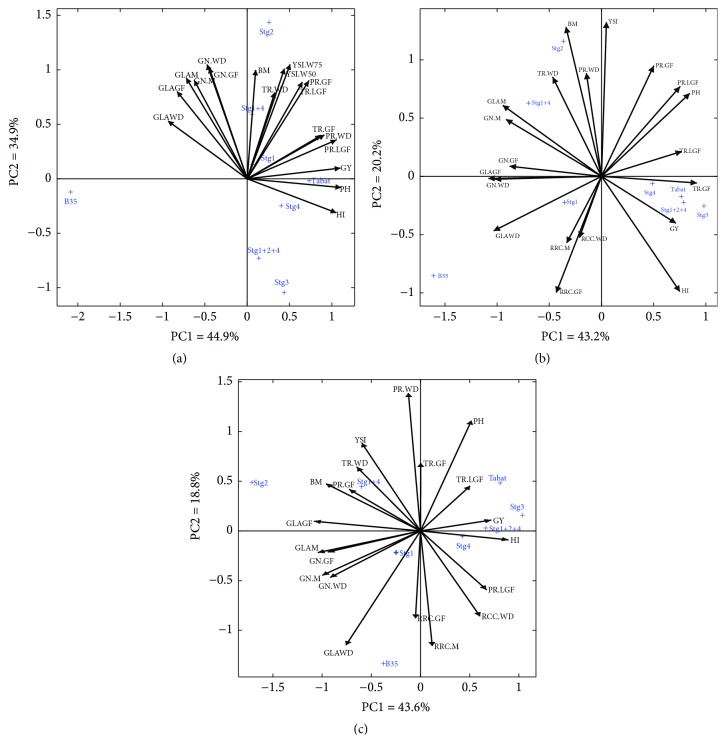
PCA analysis of stay-green and yield traits measured in six stay-green sorghum introgression lines and their parents under W_100_ (a), W_75_ (b), and W_50_ (c). Traits included in the PCA are grain yield (GY), biomass (BM), harvest index (HI), plant height (PH), yield susceptibility index (YSI), relative chlorophyll content in SPAD units (RCC), relative number of green leaves/plants (GN), green leaf area (GLA), transpiration rate (TR), and photosynthesis rate (PR). PR and TR were measured at one week before drought (WD), mid-grain filling (GF), and late-grain filling (LGF) whereas RCC, GN, and GLA were measured at WD, GF, and maturity (M).

Principal component analysis (PCA) (Figure 6) illustrates the differences between the three water regimes in traits association. Sum of PC1 and PC2 explained 79.8, 63.4, and 62.4% of the total variation in W_100_, W_75_, and W_50_, respectively (Figures 6(a), 6(b), and 6(c)). Under all treatments, PC1 showed high coordination with GY and HI, whereas PC2 showed high coordination with BM and YSI. PC2 had a negative correlation with GY. Thus, it was called stress susceptibility component. This component separated genotypes with high and low GY in different environments.

Under the W_100_ condition with no drought, GY correlated with the HI, PH, PR, and TR at WD, GF, and LGF. The stay-green traits showed negative and no association with the GY (Figure 6(a)), although YSI of W_75_ and W_50_ were strongly correlated. Under W_75_, the GY was associated with HI, PH, and PR at WD and GF, and TR at GF and LGF. There was an association between the GY and RCC at GF. GY correlated negatively with BM and YSI and the stay-green traits (GN and GLA) at all stages (Figure 6(b)). Under W_50_, the GY was associated with HI, PH, and PR at WD and LGF, TR at LGF, and the RCC at WD and GF (Figure 6(c)). On the other hand, GY was negatively associated with the BM and YSI. There was a close association between BM and stay-green trait (GN and GLA) under all drought treatments.

Selection of genotypes with high PC1 and low PC2 indicates the suitable genotypes for both stress and non-stress environments [42, 43]. Based on the PCA we classified the genotypes into four groups according to their GY under non-stress and stress conditions: genotypes with high GY under both stress and non-stress conditions (Group A), genotypes with high GY only under non-stress conditions (Group B), genotypes with high GY only under stress conditions (Group C), and at last genotypes with low GY under both conditions (Group D). Thus, Stg1+2+4 and Stg3 with rather higher PC1 and lower PC2 are superior genotypes under both stressed and non-stressed conditions (Figures 6(a), 6(b), and 6(c)). These genotypes had stable performance in the circumstances of low sensitivity to drought stress. Therefore, they belong to Group A. Stg2 could be known as Group D. This genotype is drought sensitive and had low GY and HI under drought conditions.

### 3.5. Regression Analysis

We applied regression analysis to provide more information on the significance of the important associations identified by the PCA analysis. The results indicated that HI contribution to the GY in the stay-green BILs was significant under the three water regimes (W_100_, W_75_, and W_50_) (Figure S1). The PH had a significant association with the GY only under W_100_ without drought (Figure S2). Under W_75_, PR was the most contributing factor (R^2^=0.85^*∗∗*^), whereas under W_50_, TR at GF was the most important factor contributing to GY (R^2^=0.69^*∗*^) (Figure S3). GLA at M was strongly correlated with GN (R^2^= 79^*∗∗*^, R^2^=0.87^*∗∗*^ and R^2^= 0.87^*∗∗*^) under W_100_, W_75_, and W_50_, respectively (Figure S4).

## 4. Discussion

Stay-green is positively correlated with sorghum yield under post-flowering drought stress [10]. Although a positive correlation between stay-green and GY has been demonstrated in earlier studies, the physiological and molecular basis of the stay-green trait remains unclear [30]. Our results showed that stay-green QTLs affected a number of traits under terminal drought conditions in sorghum, and the significance and magnitude of the effects depended critically on the drought severity.

### 4.1. The Drought Threshold for the Stay-Green Lines

We performed this study at three levels of soil FC, W_100_, W_75_, and W_50_. Our results indicated that the performance of the stay-green lines reduced with the reduction in soil moisture content in all traits. W_50_ was the most effective treatment in classifying and reducing the performance of the stay-green lines, and the most affected traits were the PR at LGF and GLA (Tables 1 and 2). From W_100_ to W_50_, the GY of TAB reduced by 70%, whereas that of the BILs reduced by only 36%. This finding shows clearly the effectiveness of the stay-green QTLs in enhancing the adaptation of TAB to the post-flowering drought. Interestingly, we observed clear expression and response of the stay-green trait under the moderate drought (W_75_) in terms of GLA and GN at all stages and RCC at maturity. An earlier report of Mahalakshmi and Bidinger [44] indicated that moderate but prolonged terminal drought stress during GF is the ideal environment for evaluating the stay-green trait. However, in this study we could identify that drought as 75% of the soil FC could induce the expression of the stay-green and, hence, it could be enough to evaluate genotypes for stay-green traits.

### 4.2. The Impact of the Stay-Green QTLs under Non-Stress Conditions

Understanding the effect of the stay-green QTLs in the modification of plant performance under adequate soil moisture is essential as drought is fluctuating from year to year and place to place, and thus elasticity in crop performance is essential to have good yield with less or adequate water. Our results indicated that the single stay-green QTLs have no negative impact on the GY of TAB. However, the combination of Stg1+4 showed a decrease in TAB GY under W_100_ conditions (Table 1). Other stay-green QTLs showed different impacts in TAB background; Stg1, Stg2, and Stg1+4 increased the GLA (Figure 1) and Stg3 and Stg1+2+4 decreased the TR at WD and LGF but not at the GF (Figure 5). The PCA analysis indicated that HI contributes to the GY (Figure 6(a)). Thus, we attribute the lower GY of Stg1+4 to its low HI. Borrell [30] reported that stay-green QTLs have no consistent yield penalty under irrigated conditions without drought. We attribute this contradiction of findings to the following: (1) Borrell [30] studied the effect of single QTLs only and did not study the effect of multiple QTLs, (2) there is a difference between the genetic backgrounds used in this study and that employed by Borrell [30], and (3) there is linkage drag as these lines still at BC_3_. Vadez [9] showed that the stay-green QTLs, Stg1 and Stg3, decreased the tillering and leaf area in S35 background, whereas there was no such effect in R16 background. Thus, they concluded that the impact of the stay-green QTLs depends on their interaction with the genetic background. Therefore, it is important for the breeding programs to consider this interaction to assure good performance and yield in the wet periods.

### 4.3. Association between the Studied Traits and GY under Normal and Drought Conditions

Understanding the association between the traits and the GY under different soil moisture contents is a prerequisite to decide which traits should be focused on in the breeding programs to increase the GY and also to understand how the stay-green contributes to increasing or stabilization of GY under drought conditions. Our results indicated that HI, PH, TR, and PR are the major traits contributing to the GY at all soil moisture levels (Table 1, Figure 6). Interestingly, the TR contribution to the GY differs among the treatments; it was important at the W_100_ condition and W_75_ at GF and LGF, whereas it was important at LGF at W_50_. The contribution of the PR varied with the variation in the soil moisture content. The contribution was high at the W_100_ and W_75_, whereas it was less at W_50_ (Figures 6(a), 6(b), and 6(c)). At the W_75_ and W_50_, PR at LGF was more important than at WD and GF for higher GY. These findings indicated clearly that the response of the stay-green lines to the post-flowering drought depends on the drought severity level. These findings are consistent with the findings of Vadez [9]. They concluded that variation in GY of stay-green QTLs in different genetic backgrounds was due to HI and transpiration efficiency. The difference between our study and Vadez [9] is that they estimated the transpiration efficiency by measuring the water supply and consumption, and we estimated the TR of the plants leaves by direct measurement of the leaf activity under two levels of drought at three different developmental stages. Thus, we were able to understand the change in the plant behavior with the progress of drought and development of the plant. Our findings and that of Vadez [9] indicate that the stay-green genotypes stabilize their GY under drought by manipulation of their behavior of water uptake and utilization, photosynthesis performance, and increasing the mobilization of the photo-assimilate to the grains (high HI). This manipulation of physiological performance or behavior depends on the degree of drought severity and the genetic background.

In this study, PH was associated with GY at the three water regimes (Figures 6(a), 6(b), and 6(c)). The association was high and significant under W_100_ compared to that at W_75_ and W_50_ (Figure S2). This could be explained by the findings of Sabadin [45] that PH QTLs were colocalized with the GY and stay-green QTLs. On the other hand, taking into consideration that also HI association with GY was higher under W_100_ compared to that under W_75_ and W_50_ (Figure S1), we can suggest that stay-green BILs are less reliant on the stem reserve under drought and operate another mechanism to maintain or stabilize their GY under drought. In addition, high GY could be reasonably predicted from PH (*R*^2^*=*0.92^*∗∗∗*^) under non-stress environment.

HI was correlated with RCC at WD in W_100_ and W_75_ but not in W_50_, where it was correlated with PR and RCC at GF and M (Figure 6). These results implied that remobilized reserves explained at least more than 60% of the variation observed in the GY of stay-green BILs tested under normal and moderate or severe drought condition. Ongom [46] suggested that plants with high remobilization could perform well under post-flowering drought. Under severe drought, PR and RCC at GF and M could be good indicators for high GY. Furthermore, PR had a positive association with GLA only under W_50_, and this may explain its role under severe drought and could be explained by the findings of Swain [47]; that is, variation in photosynthesis is associated with leaf protein content.

The GLA and RCC were correlated at WD, GF, and M under W_100_, W_75_, and W_50_ and were not correlated with the GY (Figure 6). These findings indicate that higher leaf senescence is due to higher translocation of food reserve from leaves to grains for better grain filling and increased GY as reported by Reddy [25]. This finding also explains the importance of the high HI trait in the stabilization of the GY under the drought conditions. This is typically the case of the BIL Stg3 as it had more HI and GY than the other BILs, but it had low GLA and RCC compared to the other BILs. Furthermore, GLA at M and GF stages were significant and positively correlated with GN under all soil moisture levels (W_100_, W_75_, and W_50_) indicating that GN can be used as easy/fast indicator for stay-green trait instead of GLA.

### 4.4. Stay-Green QTLs Contribution to the GY Stabilization under Drought

As a result of their low rate of leaf senesce, all the stay-green single QTLs improved or increased the GN under both the moderate and the severe drought (Figure 3), especially at M. Under W_75_, Stg3 and Stg4 did not affect the GLA (Figure 1), whereas at W_50_ all the Stg QTLs increased/maintained high GLA. These results indicate that the impact of the Stg QTLs depends on the degree of the drought severity. Our results indicated that Stg2 and Stg1+4 had the lowest reduction in GN and GLA, and similar results were reported by Jordan [48].

The stay-green trait is positively correlated with GY in field conditions under terminal drought [6, 8, 10, 47]. The QTLs contribution in the GY varied with a variation in the soil moisture content or, in other words, with the severity of the drought. At the W_100_ condition, none of the QTLs increased the GY in TAB, and Stg1+4 decreased the GY, whereas at W_75_ and W_50_ all the stay-green QTLs increased the GY. Stg3 and the combination of Stg1+2+4 were the most efficient QTLs in terms of GY performance. These lines decreased the TR under W_100_ condition, whereas under drought both possessed higher TR. Interestingly the high TR in these two lines was related to specific stages depending on the drought severity. At W_75_ Stg3 increased the TR at WD and LGF, whereas at W_50_ the TR was high at the GF and LGF. Stg1+2+4 increased the TR at GF under both W_75_ and W_50_ (Figure 5). In addition, these two lines possessed high HI and increased PR at GF and LGF (Table 1, Figure 5). Based on these results we attribute the high GY of Stg3 and Stg1+2+4 to their high HI, PR, and TR. Stg3 is found to be positively important for improving GY under post-flowering drought stress (Table 1), and similar results were reported by Reddy [25]. Also, Sabadin [45] pointed out the colocalization of Stg3 and GY QTL and suggested the potential of indirect selection based on stay-green to improve sorghum GY under drought.

Stg2 showed very low HI and had a great reduction in GY under W_75_ and W_50_ compared to the other Stgs (Table 1), although it has a lower reduction in GN (Table 1). This result explains that variation in GY reduction depends on the stress severity since the variation in GY/panicle was found to be a function of terminal drought [39]. Moreover, introducing Stg QTL into highly senescent background could affect the sink source relationship as reported by earlier studies of Kassahun [49]. Our results confirmed previous reports that Stg2 is an important QTL for maintaining higher GLA contributing to slow senescence (Figure 4). This QTL was also reported to contribute to higher GLA at WD and M [50] and to %GLA at 45 days after flowering [15] in different genetic backgrounds. Reddy [25] indicated that the expression of Stg2 QTL was consistent and formed an important QTL for marker-assisted improvement of post-rainy sorghum lines for terminal drought tolerance. We attributed the low GY observed for Stg2 in our study to the presence of linkage drag and the difference of the genetic background and the environments.

### 4.5. Impact of the QTL Pyramiding

In this study, we compared the effect of the single QTLs and two combinations of double and triple QTLs on the adaptation to post-flowering drought stress. The performance in terms of GY of the single QTL Stg3 under both drought treatments W_75_ and W_50_ was similar or better than that of the double or triple QTLs (Table 1). However, the effect of the QTL pyramiding was evident in the tolerance of the BILs; using the YSI we classified Stg1 and Stg4 as moderately tolerant but when these QTLs were combined in Stg1+4 the tolerance increased, and Stg1+4 was classified as tolerant. Interestingly, when the sensitive QTL Stg2 was coupled with Stg1+4 the combination Stg1+2+4 was classified as tolerant. On the other hand, Stg2 had lower GY and HI under drought, but, when combined with Stg1+4 (the combination Stg1+2+4), it increased the GY and HI under drought (Table 1). These findings indicate that QTL pyramiding can enhance the adaptation to post-flowering drought. However, this effect needs to be investigated across different backgrounds and environments, as the stay-green QTLs effect on improving adaptation to post-flowering drought was found to be dependent on the genetic background and the environment [9, 51]. In addition, in this study, the single QTL Stg3 had comparable GY to that of the combination Stg1+2+4 which suggests that QTL pyramiding might not always be necessary depending on the environment and genetic background.

### 4.6. The Putative Model of Stay-Green Adaptation to Post-Flowering Drought

Using stay-green introgression lines in the background of RTX7000, Borrell [5, 30] concluded that stay-green genotypes adapt to the post-flowering drought through decreased tillering and the size of upper leaves, which reduced canopy size at flowering. This reduction in transpirational leaf area reduced pre-flowering water demand, thereby increasing water availability during GF and, ultimately, GY. Recently, Borrell [30] using the same introgression lines reported that tiller leaf area rather than transpiration efficiency, or transpiration per leaf area, was the main driver of weekly transpiration and the reduced pre-flowering water use in stay-green lines. In this study, we did not observe any reduction in the GLA of the stay-green lines, and stay-green QTLs Stg1, Sg2, and Stg1+4 increased the GLA of the senescent parent TAB before flowering under both control and drought conditions. We attribute this contradiction in findings to the difference in the genetic backgrounds used, especially that Vadez [9] showed that the stay-green QTLs, Stg1, and Stg3 decreased the tillering and leaf area in S35 background, whereas there was no such effect in R16. This contradiction demonstrates that (1) the stay-green plants adapt to the post-flowering drought through other different mechanisms and not only GLA reduction and water saving before flowering and (2) the stay-green effect depends largely on the genetic background.

In these earlier reports, plants water utilization behavior was evaluated by measuring the plant water consumption and transpiration efficiency. In this study, we measured the actual leaf TR at three different stages under two different levels of post-flowering drought severities. This enabled us to examine in more detail the behavior of the Stg QTLs in the modification of the plant behavior under drought. Thus, based on our results and the other reports we can suggest that stay-green genotypes adapt to post-flowering drought by reducing the transpirational leaf area and the TR per leaf that reduce pre-flowering water demand, thereby increasing water availability during grain filling and utilizing the conserved water depending on the drought severity and the genetic background.

## 5. Conclusion

In conclusion, our results clearly showed that the stay-green QTLs enhance post-flowering drought response to a level up to 50% of the soil FC and the stay-green trait is expressed under the moderate drought (W_75_). The stay-green QTLs help to increase or stabilize the GY under drought through efficient water utilization (TR) depending on the drought severity coupled with the high rate of photo-assimilates translocation (high HI). QTL pyramiding could increase the drought tolerance but might not always be necessary to stabilize and increase the GY under post-flowering drought. The understanding of the physiological mechanisms associated with drought severity, senescence, and photosynthetic efficiency and the connection between QTL expression/interaction with genetic background and physiological response to drought could be the key to remove the plateau of productivity associated with sorghum adaptation to unfavorable environmental conditions.

## Figures and Tables

**Table 1:** Grain yield, percentage (%) over TAB, biomass, harvest index, and plant height of the six stay-green introgression lines and their parents evaluated under control conditions (W_100_, 100% soil field capacity) and two post-flowering drought conditions (W_75_, 75%; W_50_, 50% soil field capacity).

	Grain yield (g)			% Over TAB		Biomass (g)			Harvest index (%)			Plant height (cm)		
Genotypes	W_100_	W_75_	W_50_	W_75_	W_50_	W_100_	W_75_	W_50_	W_100_	W_75_	W_50_	W_100_	W_75_	W_50_
TAB	48.4	28.0	14.4	—	—	300.0	255.0	210.0	16.1	12.1	6.4	167.3	145.0	142.3
B35	15.3	16.6	14.2	—	—	280.0	256.0	236.0	6.1	8.9	6.1	115.7	116.7	117.0
Stg1	46.5	35.5	27.0	26.9	87.5	338.3	320.0	260.0	12.1	10.4	7.1	166.3	136.0	134.0
Stg2	44.4	13.5	3.2	-51.8	-77.9	320.0	350.0	333.3	14.0	3.8	1.2	161.7	150.3	142.3
Stg3	42.2	43.3	41.2	54.6	186.1	250.0	246.7	206.7	16.5	17.1	19.1	172.3	162.3	166.3
Stg4	41.2	33.4	29.1	19.2	102.3	275.0	290.0	270.0	15.5	10.5	7.8	160.3	158.7	146.3
Stg1+4	39.0	35.0	31.0	25.1	115.3	333.3	310.0	315.0	10.7	8.3	4.0	160.0	156.3	149.0
Stg1+2+4	43.6	42.1	32.1	50.2	122.9	280.0	276.7	213.3	14.3	14.3	14.9	162.0	163.3	160.3

Mean	40.1	30.9	24.0	—	—	297.1	288.0	255.5	13.2	10.7	8.3	158.2	148.6	144.7
SEM±	2.195	2.210	2.560	—	—	7.510	7.860	9.740	0.740	0.860	1.140	4.290	3.330	3.180
^a^LSD (^b^G)	8.880	6.180	8.180	—	—	38.060	34.840	23.690	3.2	3.1	1.2	24.9	10.2	12.0
LSD (G x ^c^T)	3.980	3.980	3.980	—	—	18.650	18.650	18.650	1.630	1.630	1.630	9.41	9.41	9.41
*P* value (G)	<0.0001	<0.0001	<0.0001	—	—	0.002	0.000	<0.0001	<0.0001	<0.0001	<0.0001	0.007	<0.0001	<0.0001
*P* value (G x T)	<0.0001	<0.0001	<0.0001	—	—	0.004	0.004	0.004	<0.0001	<0.0001	<0.0001	0.15ns^d^	0.15ns	0.15ns
CV (%)	26	35	41	—	—	12	13	18	27	39	45	13	11	10

^a^LSD: least significant difference:* P*<0.05; ^b^G: genotypes; ^c^T: drought treatment; ^d^ns: not significant.

**Table 2:** Green leaf area, relative chlorophyll content, relative number of green leaves/plants, photosynthesis rate, and transpiration rate of the six stay-green introgression lines and their parents evaluated under control condition (W_100_, 100% soil field capacity) and two post-flowering drought conditions (W_75_, 75% soil field capacity; W_50_, 50% soil field capacity).

	Green leaf area			Relative chlorophyll			Relative number of			Photosynthesis rate			Transpiration rate		
	(cm^2^)			content			green leaves/plants			(*µ*mol CO_2_ m^−2^ s^−1^)			(mmol H_2_O m^−2^ s^−1^)		
	WD	GF	M	WD	GF	M	WD	GF	M	WD	GF	LGF	WD	GF	LGF
W_100_	679^a^	585^a^	262^a^	—	—	—	50.8^a^	43.8^a^	21.0^a^	27.5^a^	27.2^a^	16.9^a^	4.0^a^	3.4^a^	2.7^a^
W_75_	619^a^	539^b^	251^a^	95.2^a^	95.2^a^	53.8^a^	45.8^b^	40.4^b^	18.5^b^	23.8^b^	21.2^b^	10.2^b^	4.1^ab^	1.4^b^	0.6^b^
W_50_	604^a^	449^c^	193^b^	92.8^b^	77.9^b^	46.4^b^	43.5^b^	33.1^c^	13.9^c^	24.9^ab^	17.6^c^	8.6^c^	4.2^b^	2.2^c^	1.8^c^

WD: one week before drought; GF: mid-grain filling; LGF: late-grain filling; M: maturity. Different letters are significantly different (Fischer PLSD, *P*<0.05).

## Data Availability

The data used to support the findings of this study are included within the article.

## Supplementary Materials

Supplementary Materials
** Figure S1.** Relationship between grain yield (GY) and harvest index (HI) for six stay-green introgression lines and their parents evaluated under W_100_ (a), W_75_ (b) and W_50_ (c) of soil field capacity. *∗* denote significant differences at* P* ≤ 0.05.** Figure S2.** Relationship between grain yield (GY) and plant height (PH) for six stay-green introgression lines and their parents evaluated under W_100_ (a), W_75_ (b) and W_50_ (c) of soil field capacity. ∗∗∗ denote significant differences at* P* ≤ 0.001, ns denote not significant.** Figure S3.** Relationship between photosynthesis rate (PR) at grain filling and grain yield (GY) under W_100_ (a), transpiration rate (TR) at grain filling and grain yield (GY) under W_50_ (b) for six stay-green introgression lines and their parents. *∗*, *∗∗* denote significant differences at* P* ≤ 0.05 and* P *≤ 0.01, respectively.** Figure S4.** Relationship between green leaf area at grain filling and maturity (GLA.GF and GLA.M, respectively) and relative number of green leaves/plant under W_100_ (a), W_75_ (b) and W_50_ (c) for six stay-green introgression lines and their parents. *∗*, *∗∗*, ∗∗∗ denote significant differences at* P* ≤ 0.05,* P* ≤ 0.01,* P* ≤ 0.001, respectively.
