# Supplementary material for: Stay-Green QTLs Response in Adaptation to Post-Flowering Drought Depends on the Drought Severity
Source: Biomed Res Int. 2018 Nov 18;2018:7082095. doi: 10.1155/2018/7082095 (PMC6280221; doi:10.1155/2018/7082095)
Supplement: Supplementary Materials — Figure S1. Relationship between grain yield (GY) and harvest index (HI) for six stay-green introgression lines and their parents evaluated under W100 (a), W75 (b) and W50 (c) of soil field capacity. ∗ denote significant differences at P ≤ 0.05. Figure S2. Relationship between grain yield (GY) and plant height (PH) for six stay-green introgression lines and their parents evaluated under W100 (a), W75 (b) and W50 (c) of soil field capacity. ∗∗∗ denote significant differences at P ≤ 0.001, ns denote not significant. Figure S3. Relationship between photosynthesis rate (PR) at grain filling and grain yield (GY) under W100 (a), transpiration rate (TR) at grain filling and grain yield (GY) under W50 (b) for six stay-green introgression lines and their parents. ∗, ∗∗ denote significant differences at P ≤ 0.05 and P ≤ 0.01, respectively. Figure S4. Relationship between green leaf area at grain filling and maturity (GLA.GF and GLA.M, respectively) and relative number of green leaves/plant under W100 (a), W75 (b) and W50 (c) for six stay-green introgression lines and their parents. ∗, ∗∗, ∗∗∗ denote significant differences at P ≤ 0.05, P ≤ 0.01, P ≤ 0.001, respectively. [file 7082095.f1.pdf]

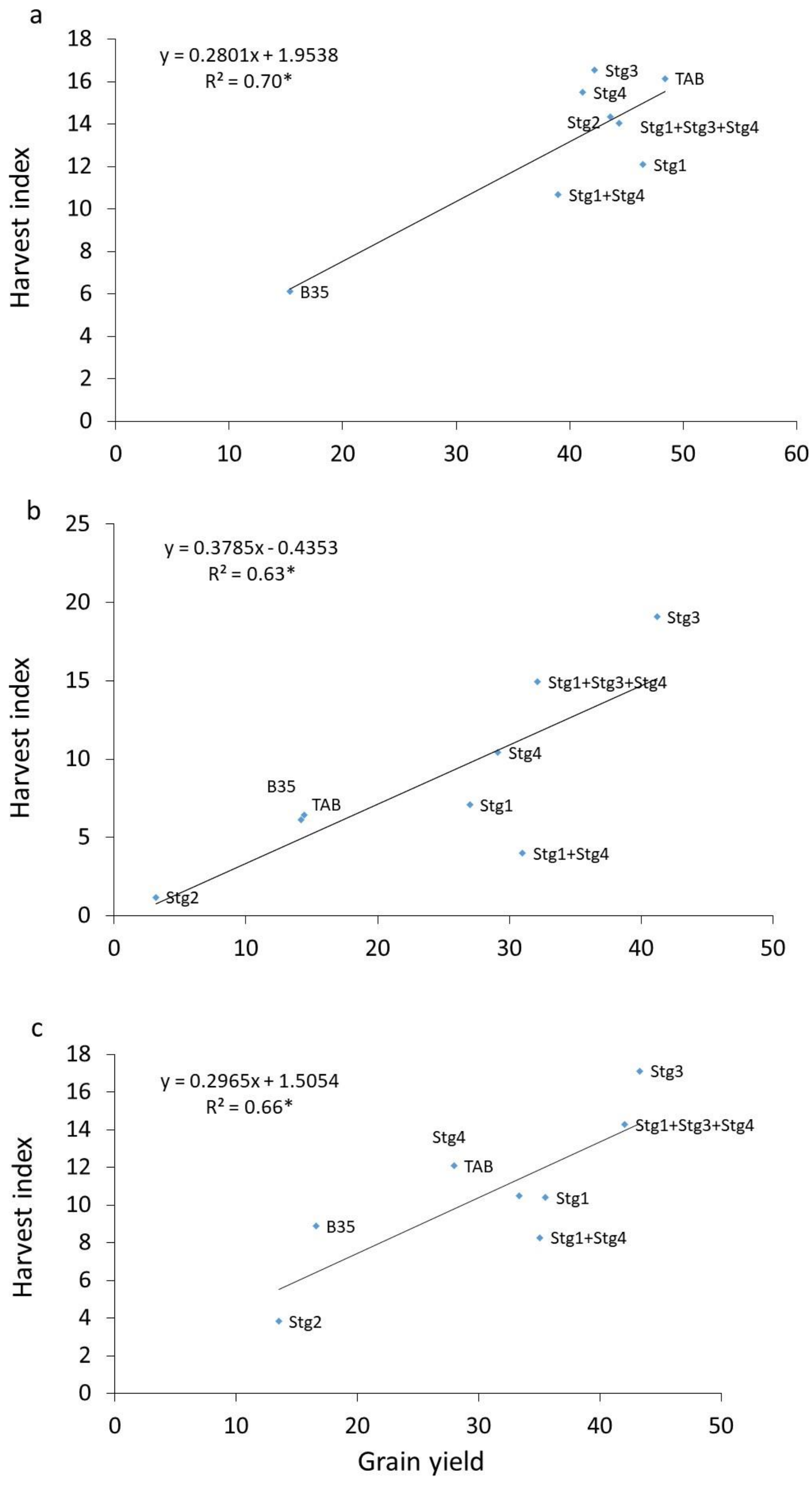

Figure S1 Relationship between grain yield (GY) and harvest index (HI) for six stay-green introgression lines and their parents evaluated under W_{100} (a), W_{75} (b) and W_{50} (c) of soil field capacity. * denote significant differences at $P \leq 0.05$.

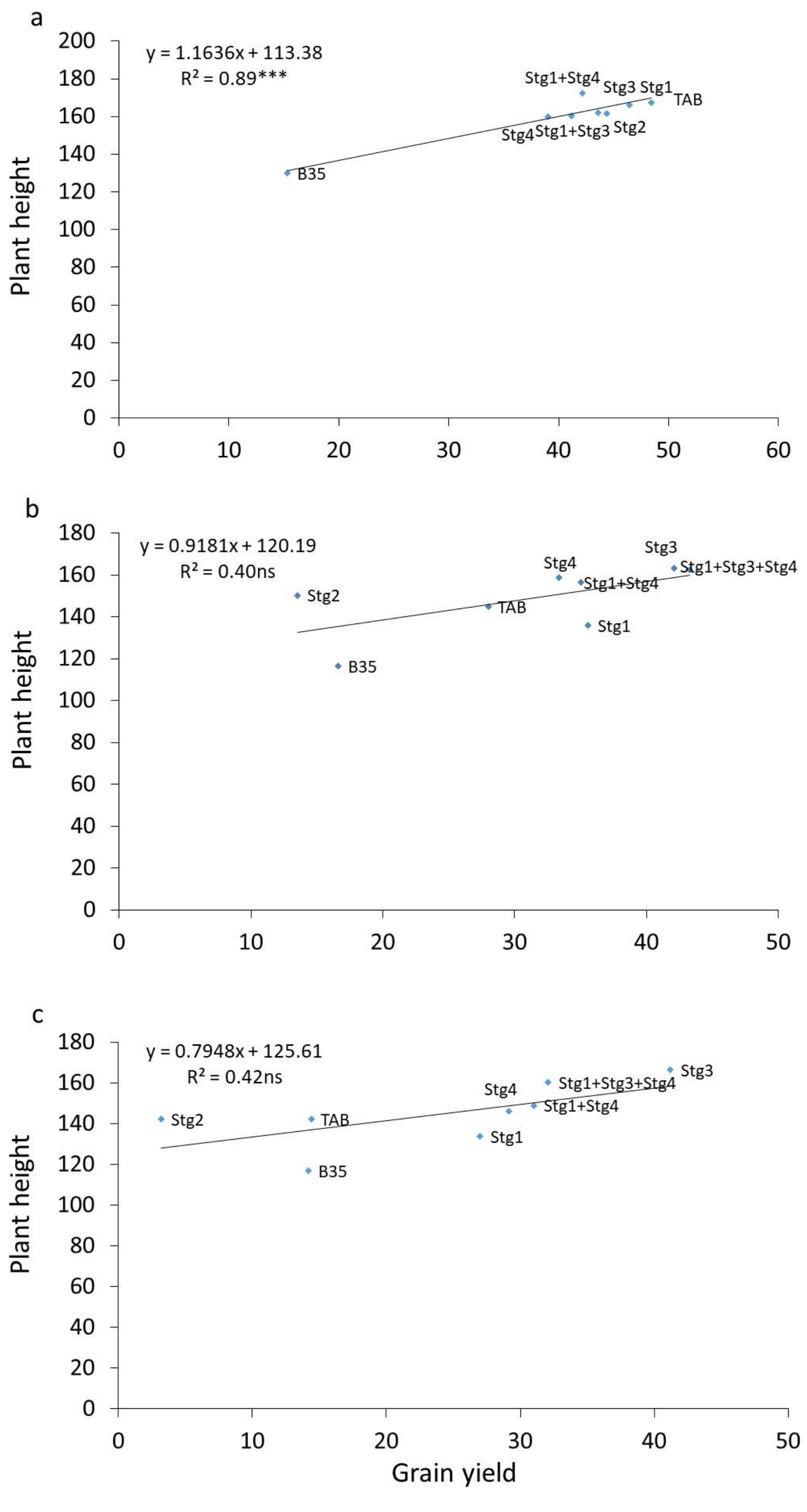

Figure S2 Relationship between grain yield (GY) and plant height (PH) for six stay-green introgression lines and their parents evaluated under W_{100} (a), W_{75} (b) and W_{50} (c) of soil field capacity. *** denote significant differences at $P \leq 0.001$, ns denote not significant.

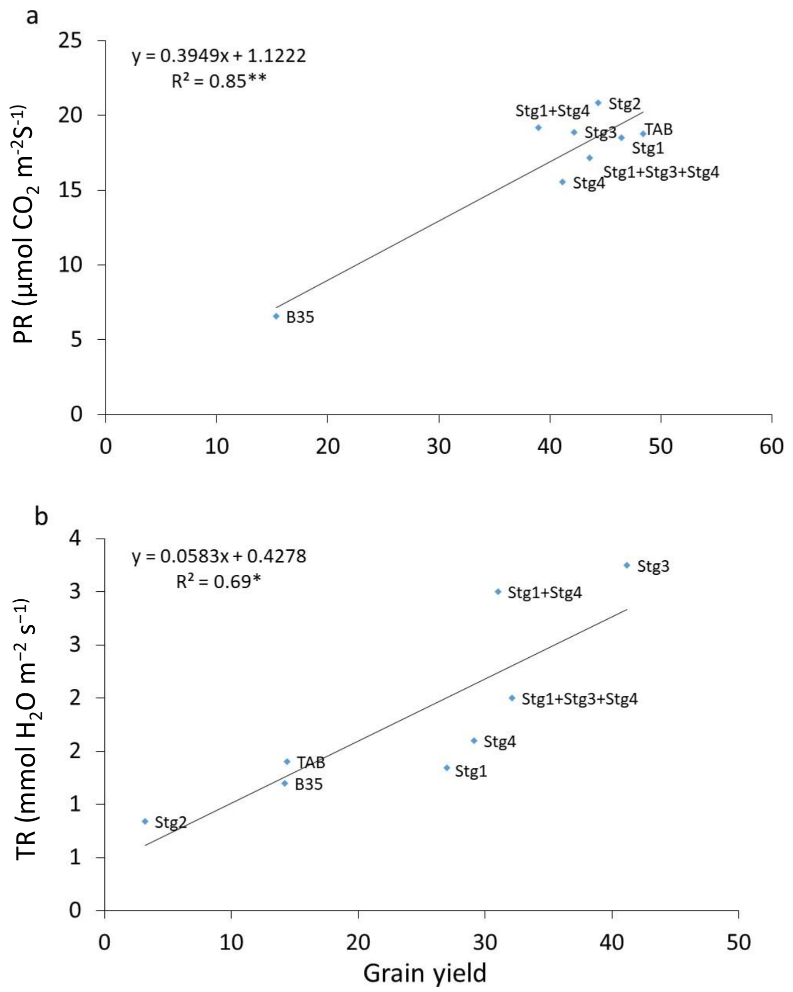

Figure S3 Relationship between photosynthesis rate (PR) at grain filling and grain yield (GY) under W_{100} (a), transpiration rate (TR) at grain filling and grain yield (GY) under W_{50} (b) for six stay-green introgression lines and their parents. *, ** denote significant differences at $P \leq 0.05$ and $P \leq 0.01$, respectively.

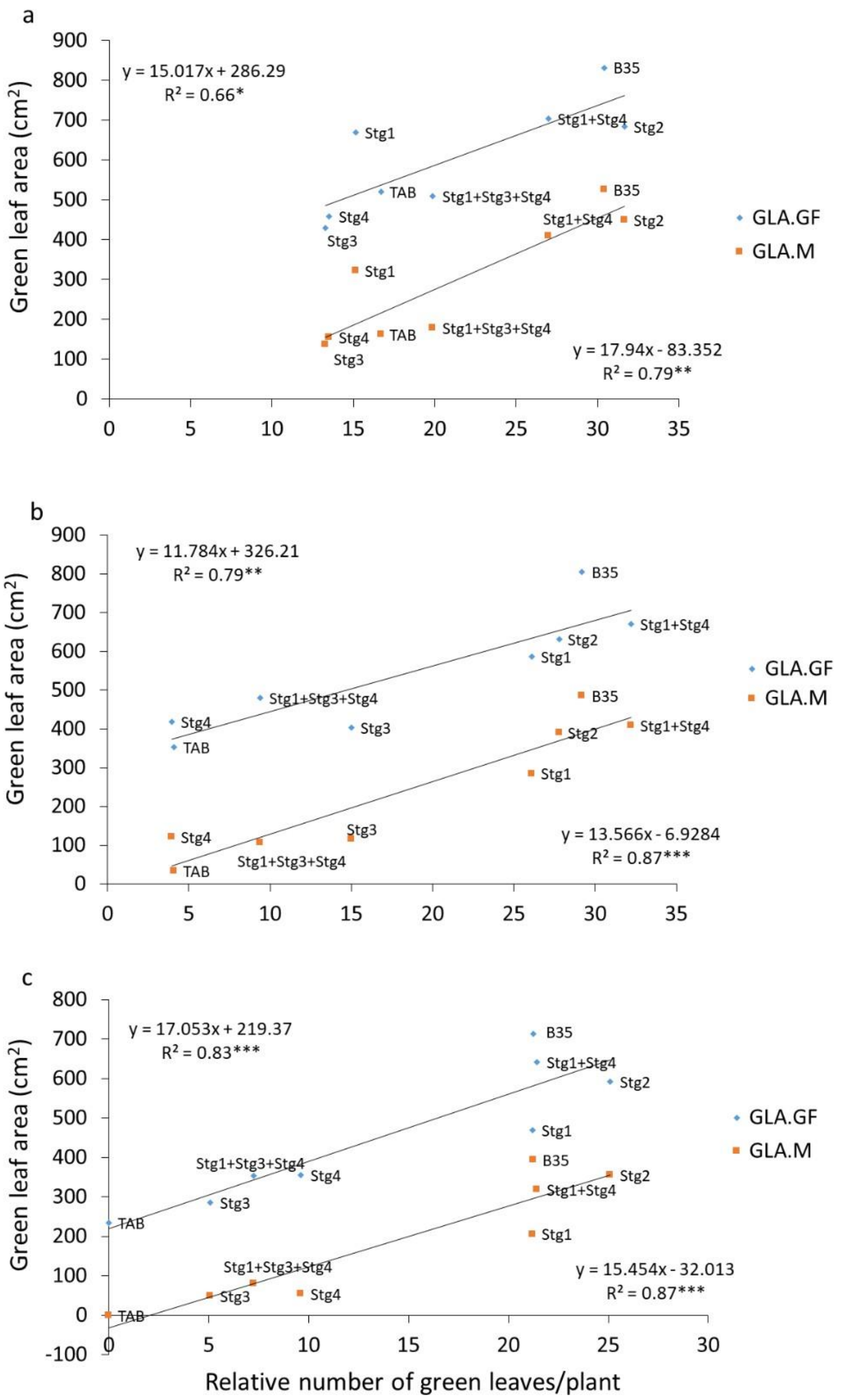

Figure S4 Relationship between green leaf area at grain filling and maturity (GLA.GF and GLA.M, respectively) and relative number of green leaves/plant under W_{100} (a), W_{75} (b) and W_{50} (c) for six stay-green introgression lines and their parents. *, **, *** denote significant differences at $P \leq 0.05$, $P \leq 0.01$, $P \leq 0.001$, respectively.
